# Range-wide genetic population structure of common pochard (*Aythya ferina*): a potentially important vector of highly pathogenic avian influenza viruses

**DOI:** 10.1002/ece3.46

**Published:** 2011-12

**Authors:** Yang Liu, Irene Keller, Gerald Heckel

**Affiliations:** 1 Computational and Molecular Population Genetics, Institute of Ecology and Evolution, University of BernBaltzerstrasse 6, 3012, Bern, Switzerland; 2 Swiss Institute of BioinformaticsGenopode, 1015 Lausanne, Switzerland; 3 Department of Aquatic Ecology and Department of Fish Ecology and EvolutionEawag, Überlandstrasse 133, 8600, Dübendorf, Switzerland

**Keywords:** Diving duck, H5N1, Microsatellites, Migration, Mitochondrial DNA, Population genetics

## Abstract

An understanding of the distribution and spatial structure of the natural vectors of zoonothic pathogens is of interest for effective disease control and prevention. Here, we investigate the range-wide population genetic structure of common pochard (*Aythya ferina*), a long-distance migratory duck and potential vector of highly pathogenic avian influenza. We collected several hundred samples from breeding and wintering grounds across Eurasia including some H5N1-positive individuals and generated partial sequences of the mitochondrial control region and multilocus microsatellite genotypes. Genetic differentiation among breeding populations was significant for both marker types but higher for maternally inherited mtDNA than for biparentally inherited nuclear markers. There was only weak genetic divergence between ducks sampled in Europe and East Asia, and genetic differentiation between populations was not generally associated with geographical distance. No evidence of genetic substructure was detected for ducks sampled on the European wintering grounds. Our results suggest limited breeding-site fidelity, especially in females, but extensive population admixture on the wintering grounds. The specific role of pochards as natural vectors of zoonotic pathogens and in particular H5N1 remains to be clarified but our results point to wintering grounds as potential hotspots for disease transmission.

## Introduction

Zoonoses are diseases that are transmitted from animals to humans (Jones et al. 2008). Most zoonoses (71.8%) originate in wildlife, and the incidence of zoonotic events has globally increased over recent time (Jones et al. 2008). Apart from being sources of zoonotic pathogens, wild animals can additionally play a significant role in the geographic spread of pathogens (Morens et al. 2004). Information about the distribution, abundance, and spatial structure of wild animal species may therefore be essential for effective disease control, especially in situations where the transmission of pathogens can be attributed to particular vector species (Kurtenbach et al. 2006). In most wild organisms, populations are spatially structured because of limited dispersal abilities or substantial natural or anthropogenic barriers to movements (e.g., rivers, mountains, habitat fragmentation), and the distribution and dynamics of associated pathogens may, in turn, be spatially heterogeneous (Biek and Real 2010). It is therefore not surprising that an increasing number of studies has taken into account the spatial organization of vector populations in order to understand patterns of infectious disease prevalence and transmission across different geographic scales (Biek and Real 2010, and examples therein).

Population genetics approaches provide a powerful toolbox for characterizing patterns of population structure in vector species and relating them to the spatial (and temporal) dynamics of associated pathogens (e.g., Kempf et al. 2009; Cullingham et al. 2010; Schmidt-Chanasit et al. 2010). In particular, it may be possible to identify certain geographic regions as potential transmission hotspots where different host populations come into contact. For example, the joint analysis of molecular and ringing data showed that northern pintail (*Anas acuta*) wintering in Japan and North America, respectively, share breeding areas in eastern Russia (Flint et al. 2009). This specific population structure thus points to a potential pathway for the exchange of pathogens between Asia and North America (Flint et al. 2009). At a more local scale, the identification of heterogeneous patterns of dispersal and gene flow among host populations may provide important information about environmental, ecological, and social factors affecting pathogen transmission. As an example, a relatively strong degree of female philopatry was shown by comparing genetic variation of maternally inherited mitochondrial DNA (mtDNA) and biparentally inherited microsatellite markers in white-tailed deer (*Odocoileus virginianus*), a vector of chronic wasting disease (Cullingham et al. 2010). Consequently, matrilineal social cohesion and social interactions among related females in the vector species may have significant effects on local transmission and dynamics of the disease (Cullingham et al. 2010).

**Figure 1:**
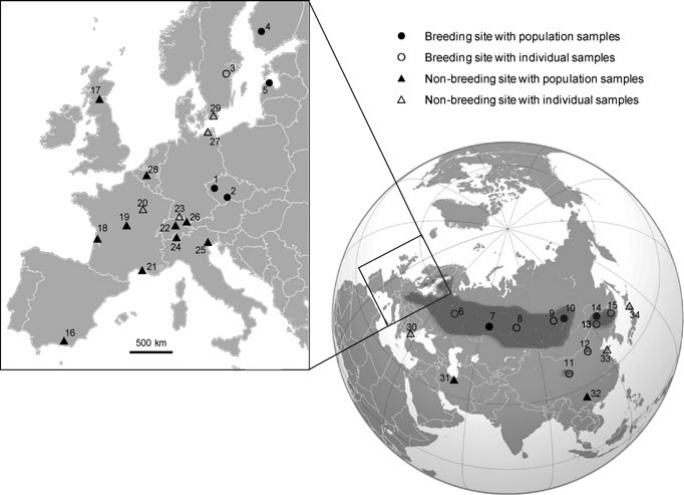
Sampling localities of the common pochard (*Aythya ferina*) across its distribution range in Eurasia. The species’ breeding range is highlighted in dark gray in the global view. Circles and triangles represent samples from the breeding and the nonbreeding colonies, respectively. Filled symbols indicate sites used for population genetic studies with sample size *N*≥ 9 at one of the marker sets and open ones represent locations with lower sample sizes used for phylogeographic analyses only. Coordinates and sample sizes for each site are displayed in Table A1.

Waterbirds have long been identified as natural reservoirs of various avian influenza (AI) virus subtypes (Webster et al. 1992), and they probably played a role in the spatial spread of highly pathogenic avian influenza (HPAI) H5N1 from south east Asia into Europe (Kilpatrick et al. 2006; Feare 2007; Starick et al. 2007). Common pochards (*Aythya ferina*, pochards hereafter) have been listed as a high-risk species in relation to AI by the European Union (2005) because they were repeatedly detected as carriers of H5N1 among a number of wild bird species. During the H5N1 outbreak in winter 2005/2006, pochards were either ranked first or second among wild bird species in terms of the number of birds found infected in France, Germany, and Switzerland (Starick et al. 2007; Hars et al. 2008; Baumer et al. 2010). The species is reported as sensitive to infection with HPAI viruses, and although the course of the infection varies between individuals (Keawcharoen et al. 2008), the discovery of an asymptomatic live H5N1-positive wild pochard in Switzerland in winter 2008 increased further the importance of the species for HPAI surveillance programs (Baumer et al. 2010). The very high abundance of pochards (Scott and Rose 1996) and their largely east–west oriented migration direction across Eurasia (Fig. 1) are of additional relevance in the context of AI surveillance in Europe. Against this background, it seems thus relevant to understand the global population structure of pochard and in particular the extent of mixing between East Asian and European ducks.

Based on ring recovery data, Blums and Baumanis (1990) distinguished four geographical subpopulations of pochard, associated with different wintering grounds: (1) Japan, (2) Eastern Asia, (3) Caspian Sea and (4) Europe. Within Europe, a further subdivision has been proposed between a northwestern population wintering around the North Sea and a southeastern population wintering in central Europe and around the Mediterranean and Black Seas (Monval and Pirot 1989; Rose and Scott 1994). However, ring recovery data indicate that the two wintering populations overlap along the North Sea coast (Keller et al. 2009), and that individuals wintering in NW or SE Europe, respectively, may breed in the same areas extending into Western Siberia (Hofer et al. 2006). The situation on breeding grounds further east is relatively unclear, and it is possible that the birds wintering in different parts of Asia may breed at different average longitudes.

In this study, we use molecular data to describe the range-wide population structure of pochards. Our primary aim was to delineate the patterns of genetic differentiation between ducks on the breeding and wintering grounds across Eurasia, with a particular focus on assessing the previously postulated subdivisions. We further examine the congruence between nuclear and mitochondrial markers with different modes of inheritance to investigate sex-specific differences in the rates of gene flow (Prugnolle and de Meeus 2002). These analyses may further help to understand the interactions between population structure of an avian host and the potential spread of zoonotic pathogens across a wide geographical range.

## Materials and Methods

### Sample collection and DNA extraction

Samples were collected from across the entire Eurasian range of pochard during our field expeditions to the Baltic States, Russia, and China between 2008 and 2010, and through ringing schemes and hunting associations between 2006 and 2010. We collected mostly feathers in a noninvasive manner, but also some muscle, blood, or egg membrane samples. A few tissue samples were obtained from museum collections. In addition, samples of individual ducks identified as H5N1-positive were available from the AI reference laboratories in Switzerland (three individuals), and Germany (five individuals). In total, 345 samples were available from 34 localities in 16 countries (Fig. 1; Table A1). Localities with *N*≥ 9 for mitochondrial and/or nuclear markers were defined as population samples and included in all population genetic analyses while samples from other localities were included in the phylogeographic analyses only. Samples collected between June and August were considered to be from breeding populations, samples collected between November and February from wintering populations. For the breeding season, we obtained seven population samples plus smaller samples from eight additional locations. On the wintering grounds, population samples were obtained from 12 sites and additional samples from another four sites (Table A1). Three small samples were considered to be from migrating individuals (combined with winter samples as nonbreeding in Fig. 1). Genomic DNA was extracted using a standard phenol–chloroform extraction protocol (Sambrook et al. 1989).

### Mitochondrial DNA sequencing

A fragment of the mitochondrial control region (*ctr*), including most of the hypervariable region (HVR) I, was amplified using the primers M1 (Sorenson and Fleischer 1996) and H774 (Sorenson et al. 1999). PCR amplification was performed in a GeneAmp® PCR System 9700 (Applied Biosystems) in a reaction volume of 25 μl, containing 12.5 μl dH_2_O, 4.8 μl dNTPs (2.5 mM), 1 μl MgCl_2_ (25 mM), 1 μl of each primer (10 pmol/μl), 2.5 μl 10× buffer (containing 1.5 mM MgCl_2_), and 0.2 μl *Taq* polymerase (5 unit/μl; QIAGEN) and 2 μl template DNA (20–100 ng/μl). The PCR amplification profile included an initial denaturation step of 93°C for 2 min, followed by 35 cycles of 93°C for 30 sec, 56°C for 30 sec, and 72°C for 1 min, and a final extension step of 72°C for 7 min. PCR products were purified with the GenElute™ PCR clean-up kit (Sigma) according to the manufacturer's protocol. Sequencing reactions were carried out in a volume of 10 μl with the Terminator Ready Reaction Mix “Big Dye” (v.3.1, Applied Biosystems) according to the guidelines of the manufacturer. The cycling protocol was as follows: 50 sec at 96°C, followed by 35 cycles of 10 sec at 96°C, 10 sec at 50°C, and 4 min and 30 sec at 60°C. The products were purified using sodium acetate precipitation, and were separated and detected on an ABI Prism 3100 Genetic Analyzer (Applied Biosystems).

Nuclear pseudogenes of mitochondrial origin (numts) are a known issue in some diving ducks, including the genus *Aythya* (Sorenson and Fleischer 1996), and may cause problems in phylogenetic analysis (Zhang and Hewitt 1996). Here, we used primer M1 that is expected to amplify only the mitochondrial target in pochard (Sorenson and Fleischer 1996). Second, we excluded a few samples (five each from CZSb and SPSe) from further analyses for which multiple fragments were amplified (assessed on minigels) or for which the final sequence showed ambiguous nucleotides. This was mainly observed in blood samples, and may be related to the relatively high ratio of nuclear to mitochondrial DNA in the nucleated red blood cells of birds (Sorenson and Quinn 1998). Finally, we carefully examined all sequences by comparing the levels of variability to reference sequences of *ctr* from pochard reported in Sorenson and Fleischer (1996) and Sorenson (pers. comm.). Because of the above restrictions, we are confident that the sequences retained for further analyses are authentic mtDNA sequences.

### Microsatellite genotyping

All samples were screened at 14 autosomal microsatellite loci initially developed for related species (Fields and Scribner 1997; Maak et al. 2003; Paulus and Tiedemann 2003; Stai and Hughes 2003; Denk et al. 2004; Hefti-Gautschi and Koller 2005; Huang et al. 2006). The 14 microsatellite loci were arranged into two multiplex reactions (Table A2) and the 5′-end of each reverse primer was modified with a pig-tail extension to facilitate genotyping (Brownstein et al. 1996). Amplification was carried out in a 10-μl reaction volume containing 5 μl of PCR mix (QIAGEN Multiplex Kit), 1 μl of a primer mix (Table A2), and 1 μl of template DNA. The PCR conditions were as follows: initial denaturation at 95°C for 15 min, followed by 30 cycles of 94°C for 30 sec, 57°C for 90 sec, and 72°C for 60 sec, and a final extension at 60°C for 15 min. Products were separated and detected on an ABI Prism 3100 Genetic Analyzer (Applied Biosystems). Fragment length was determined in comparison to an internal size standard (GeneScanTM-500LIZ™, Applied Biosystems) using GeneMapper software v.3.7 (Applied Biosystems). Approximately 15% of the samples were re-amplified and genotyped independently to ensure genotyping repeatability (Schweizer et al. 2007).

### Genetic diversity indices

The mtDNA sequences were aligned using the CLUSTALW algorithm (Thompson et al. 1994) implemented in the program BioEdit v.7.0 (Hall 1999), and were revised manually. The average number of pairwise nucleotide differences (*k*), the number of haplotypes (*N*_H_), haplotype diversity (*H*), and nucleotide diversity (π) were calculated for each population sample using DnaSP v.5.0 (Librado and Rozas 2009). For each microsatellite locus, we calculated allelic richness (*A*_R_), number of alleles (*N*_A_), and observed (*H*_O_) and expected heterozygosities (*H*_E_) in each population sample with FSTAT v.2.9.3.2 (Goudet 2002). The same program was used to test for deviations from Hardy–Weinberg equilibrium (HWE) and genotypic equilibrium, and to calculate *F*_IS_ across loci for each population and assess its significance based on 10,000 permutations. Significance levels were adjusted for multiple testing using the Bonferroni procedure (Rice 1989).

### Phylogeographic analyses based on all samples

Phylogenetic relationships among *ctr* haplotypes were reconstructed using Bayesian inference in MrBayes v.3.1.2 (BI; Ronquist and Huelsenbeck 2003). The Hasegawa–Kishino–Yano model assuming a gamma-shaped distribution across sites with an estimated proportion of invariant sites was selected as the best-fitting nucleotide substitution model based on the Akaike Information Criterion in jModelTest v.0.1.1 (Posada 2008). Four independent chains with default heating temperatures were run for 10 million steps and sampled every 1000th step. The first 25% of samples were discarded as burn-in. Convergence of the chains was assessed with the web-based program AWTY (Nylander et al. 2008). The trees were rooted using a homologous sequence from tufted duck (*A. fuligula*; Liu et al. unpubl. ms.). Further, haplotype networks were constructed based on a median-joining algorithm (Bandelt et al. 1999) in Network v.4.516 (http://www.fluxus-engineering.com). The phylogeographic structure at the nuclear microsatellite markers was investigated with principal coordinates analyses (PCoA) based on pairwise Euclidian distances between individual genotypes in GENALEX v.6.2 (Peakall and Smouse 2006), again separately for breeding and wintering birds.

### Population genetic analyses

All population genetic analyses were carried out separately for the seven breeding populations and the 12 wintering populations. Analyses of molecular variance (AMOVA; Excoffier et al. 1992) implemented in Arlequin v.3.5 (Excoffier and Lischer 2010) were performed for both marker sets to assess the proportion of genetic variance explained by the hypothesized migratory divides among breeding or wintering grounds (breeding populations: Europe, Western Siberia, Eastern Siberia, China; wintering populations: SE Europe, NW Europe, Caspian Sea, Eastern Asia). For mtDNA, we calculated pairwise Φ_ST_ with the Tamura–Nei model (Tamura and Nei 1993) and for the microsatellite data, pairwise *F*_ST_ using the estimator of Weir and Cockerham (1984) in Arlequin. Significance was assessed based on 10,000 permutations, with significance levels adjusted for multiple testing using the Bonferroni procedure. Preliminary tests showed that our main results are unlikely to be biased by temporal effects. Specifically, the patterns of genetic differentiation observed between samples collected in the same year were consistent with the results of the full analyses (data not shown). For breeding populations only, we used Mantel tests in GENALEX to test for associations between genetic distance (Φ_ST_ or *F*_ST_) and geographical distance in kilometers between pairs of populations. The significance of the association was assessed based on 1000 permutations.

We also used individual-based clustering approaches for the microsatellite data to investigate the genetic structure among breeding and wintering ducks. For Bayesian analyses with STRUCTURE v.2.3.1 (Pritchard et al. 2000), we used an admixture model with correlated allele frequencies (Falush et al. 2003) and a burn-in of 100,000 iterations followed by 500,000 Markov Chain Monte Carlo steps. The number of clusters (*K*) was varied between 1 and 7 for breeding and 1 and 12 for wintering ducks, respectively, and we carried out 10 independent runs per *K*. The most likely *K* was assessed as suggested by Evanno et al. (2005) using STRUCTURE Harvester v.0.56 (Earl 2009). We further used Discriminant Analysis of Principal Components (DAPC), a model-free multivariate method to identify genetic clusters when prior grouping information is lacking (Jombart et al. 2010). We performed DAPC and graphically displayed our results using the package “adegenet” (Jombart 2008) in R version 2.12.0 (R Development Core Team 2008). In all analyses, 33 principal components (PCs) were retained in the data transformation step, which accounted for more than 90% of the total genetic variability. The inference of the most likely number of clusters was based on the Bayesian Information Criterion (BIC; Schwarz 1978).

To assess the statistical power of our microsatellite markers to detect given levels of population differentiation, we performed power analyses in POWSIM v.4.0 (Ryman and Palm 2006). We generated 1000 datasets each for six predefined levels of population differentiation (*F*_ST_ = 0.001, 0.0025, 0.005, 0.01, 0.02, and 0.025) with samples sizes, numbers of markers, and allele frequencies corresponding to the empirical data. Statistical power was defined as the proportion of times the null hypothesis of equal allele frequencies across populations was rejected using a chi-square test or a Fisher's exact test.

## Results

### Genetic diversity

Sequences of 601 bp from the mitochondrial control region were obtained for 274 individuals, and 143 haplotypes were identified based on 80 polymorphic sites. Forty-seven haplotypes were shared by two to 36 individuals and the remaining 96 were singletons. The average number of pairwise nucleotide differences (*k*) was similar in breeding and wintering populations (4.4 and 4.38; Mann–Whitney U test, *P* = 0.96).

Microsatellite genotypes were obtained for 345 individuals. Loci Sfiu3 and MM07 were monomorphic across all populations and not considered for statistical analyses. At the remaining loci, the total number of alleles ranged from 4–17. The mean allelic richness per population was between 3.09 and 3.70 (Table 1), with no significant differences between breeding and wintering populations (Mann–Whitney *U* test, *P* = 0.14). There was no evidence of genotypic disequilibrium after Bonferroni correction. Significant heterozygote deficits were observed in 17 of 228 locus-specific tests with locus Caud13 being affected six times (Table A3). Exclusion of this locus from further statistical analyses had no significant quantitative or qualitative effect (results not shown). *F*_IS_ ranged from –0.01 to 0.22, and values were not significantly different from zero except for one population from the Czech Republic and one from Russia (Table 1).

**Table 1:** Estimates of genetic variability in 19 populations of common pochard analyzed for the mitochondrial control region and 12 microsatellite loci. The number of individuals analyzed for mtDNA (*N*_mt_) and nuclear DNA (*N*_nuc_) are given. Map ref. corresponds to the numbers provided in Figure 1, and bold font indicates breeding populations. For mtDNA, the average number of nucleotide differences (*k*), the number of haplotypes (*N*_H_), haplotype diversity (*H* ± *SD*), and nucleotide diversity (*π± SD*, in percent) were calculated. For microsatellites, mean allelic richness (*A*_R_), mean observed (*H*_O_), and mean expected (*H*_E_) heterozygosity were quantified. Multilocus inbreeding coefficients (*F*_IS_) were calculated for each population, and values in bold indicate significant deviations from Hardy–Weinberg equilibrium (HWE) after Bonferroni correction

		Mitochondrial DNA					Microsatellites				
			
Map ref.	Location	*N*_mt_	*K*	*N*_H_	*H* ± *SD*	*π± SD* (%)	*N*_nuc_	*A*_R_	*H*_O_	*H*_E_	*F*_IS_
**1**	CZSb	24	4.71	19	0.98 ± 0.02	0.80 ± 0.01	29	3.64	0.48	0.51	0.06
**2**	CZDi	9	4.44	9	1.00 ± 0.05	0.75 ± 0.13	20	3.39	0.49	0.57	**0.13**
**4**	FISo	9	7.40	5	0.81 ± 0.12	1.25 ± 0.18	9	3.11	0.47	0.56	0.18
**5**	LAKa	12	2.53	7	0.83 ± 0.10	0.42 ± 0.11	16	3.56	0.47	0.50	0.07
**7**	RUYe	7	2.29	4	0.71 ± 0.18	0.39 ± 0.15	9	3.70	0.47	0.60	**0.22**
**10**	RUBl	21	4.26	15	0.92 ± 0.05	0.72 ± 0.10	21	3.43	0.43	0.49	0.12
**14**	CNLf	16	5.17	14	0.98 ± 0.04	0.87 ± 0.10	12	3.54	0.49	0.53	0.05
16	SPSe	6	5.93	6	1.00 ± 0.10	0.99 ± 0.24	11	3.26	0.60	0.63	0.06
17	UKLe	10	5.60	9	0.98 ± 0.05	0.94 ± 0.14	10	3.67	0.52	0.54	0.05
18	FRSa	12	4.64	12	1.00 ± 0.03	0.77 ± 0.13	13	3.30	0.49	0.52	0.05
19	FRIn	16	4.23	14	0.98 ± 0.03	0.71 ± 0.12	16	3.27	0.52	0.53	0.01
21	FRCa	7	2.48	6	0.95 ± 0.10	0.41 ± 0.10	10	3.2	0.5	0.54	0.08
22	CHOb	24	3.94	16	0.95 ± 0.03	0.66 ± 0.08	67	3.47	0.51	0.54	0.05
24	ITVa	10	3.44	9	0.98 ± 0.05	0.58 ± 0.10	9	3.48	0.56	0.6	0.06
25	ITVe	13	4.59	12	0.99 ± 0.04	0.76 ± 0.08	12	3.48	0.58	0.65	0.09
26	DEBs	6	4.60	6	1.00 ± 0.10	0.77 ± 0.09	10	3.09	0.48	0.56	0.12
28	BEBr	10	3.27	8	0.96 ± 0.10	0.55 ± 0.13	10	3.35	0.51	0.54	0.06
31	IRCs	9	4.56	7	0.94 ± 0.07	0.77 ± 0.10	10	3.18	0.48	0.48	−0.01
32	CNCh	21	5.29	16	0.97 ± 0.09	0.90 ± 0.09	21	3.45	0.53	0.56	0.02

### Phylogeographic inference

**Figure 2:**
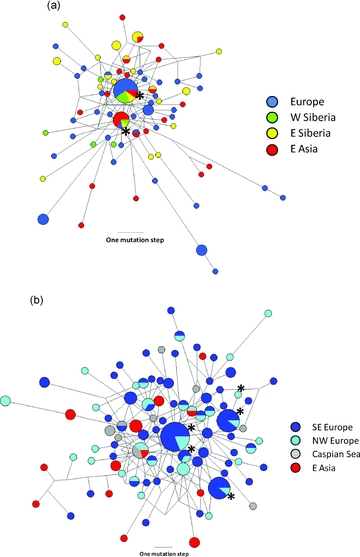
Unrooted median joining networks based on 601 bp sequences of the mitochondrial control region for (A) breeding (*N* = 119) and (B) wintering (*N* = 155) pochards. Samples from the breeding season were grouped as Europe, Western Siberia (west of Ural Mountains), Eastern Siberia, and Eastern Asia, following the population subdivision proposed by Blums and Baumanis (1990). Wintering ducks were partitioned into hypothetical subpopulations in southeastern and northwestern Europe (Scott and Rose 1996), around the Caspian Sea and in Eastern Asia. The size of the circles is proportional to the number of individuals with a particular haplotype. Asterisks mark those haplotypes that were found in H5N1-positive pochards sampled on European wintering grounds (see text).

**Figure 3:**
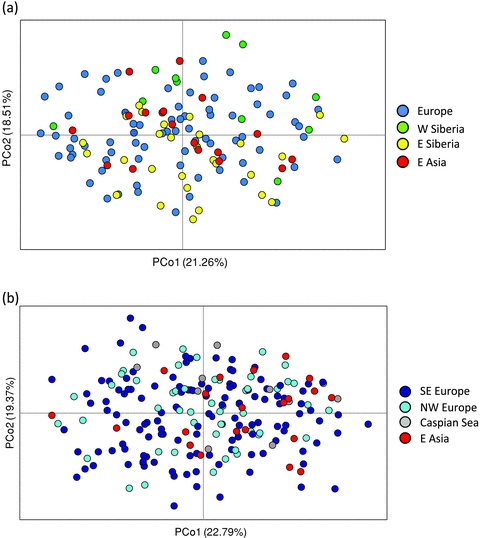
Plots of the first two coordinates from a principal coordinates analysis based on individuals genotyped at 12 microsatellite loci for (A) breeding individuals (*N* = 134) and (B) wintering individuals (*N* = 211) of pochard. Different colors represent postulated populations.

The haplotype networks show relatively little molecular differentiation in ducks sampled across Eurasia with most haplotypes separated by one to four mutational steps, and only a few unique haplotypes differing from the most frequent haplotypes by seven to nine mutational steps (Fig. 2). A few frequent haplotypes were shared by individuals from different breeding or wintering regions (Fig. 2), suggesting that no prominent genetic subdivision is associated with the major geographic regions sampled for breeding or wintering pochards. H5N1-positive individuals from Central European wintering grounds showed mostly frequent haplotypes that were shared with ducks sampled on different breeding grounds throughout Eurasia. Phylogenetic trees were star-like and did not show clusters containing individuals of a common geographical origin. Polytomies were frequent and only a few terminal nodes were well supported (posterior probability > 90%; trees not shown). Plots of the first two axes from the principal coordinates analysis based on individual microsatellite genotypes did not reveal distinct groups for breeding or wintering birds (Fig. 3). In both cases, large overlap was observed between individuals from the different geographic areas.

### Genetic population structure across Eurasia

#### Breeding populations

The population-based analyses provided no evidence of major migratory divides across Eurasia. The four geographical groups (Europe, Western Siberia, Eastern Siberia, Eastern Asia) did not explain a significant proportion of the genetic variation for mtDNA or microsatellites (Table 2). The variation attributed to differences between populations within regions, however, was significant for both marker types but larger for mtDNA (mtDNA: 8.76%, *P* < 0.0001; microsatellites: 1.74%, *P* = 0.016). For mtDNA, five of 21 pairwise comparisons between populations were significant (Table A4), and these involved two far eastern populations (RUBl & CNLf) and two European populations (CZSb & FISo). There was no evidence that pairwise genetic distances (Φ_ST_) increased with geographical distance between sampling locations (*R*^2^ = 0.034, *P* = 0.26). Based on the microsatellite markers, we obtained a global *F*_ST_ estimate of 0.018 (*P* < 0.0001), and five of 21 pairwise *F*_ST_ values were significant (Table A4). These comparisons mainly involved populations from Russia (RUYe & RUBl) and Latvia (LAKa), and two populations from the Czech Republic (Table A4). Again, there was no evidence that pairwise genetic differentiation increased with geographical distance (*R*^2^ = 0.005, *P* = 0.42).

**Table 2:** Hierarchical analyses of molecular variance (AMOVA) based on mtDNA and microsatellite loci for common pochard. Breeding populations (top) were grouped by four regions (Europe, Western Siberia, Eastern Siberia, and Eastern Asia). Wintering ducks (bottom) were partitioned according to four major wintering grounds in northwestern and southeastern Europe, around the Caspian Sea, and in Eastern Asia. Numbers in brackets represent the map reference numbers (shown in Fig. 1 and Table 1) of the populations within each group

Grouping	Markers	Variation among groups (%)	Variation among populations within groups (%)	Variation within populations (%)
[Europe (1, 2, 4, 5)]	mtDNA	−0.66	8.76**	91.90
[Western Siberia (7)]	Microsatellites	0.09	1.74*	98.17
[Eastern Siberia (10)]				
[Eastern Asia (14)]				
[Northwestern Europe (17, 18, 19, 28)]	mtDNA	1.83	0.70	97.48
[Southeastern Europe (16, 21, 22, 24, 25, 26)]	Microsatellites	0.33	0.61	99.06
[Caspian Sea (31)]				
[Eastern Asia (32)]				

* *P* < 0.05

** *P* < 0.01.

Individual-based clustering approaches provided no evidence of genetic substructuring among ducks from breeding populations. STRUCTURE analyses showed highest support for one genetic cluster across Eurasia (Fig. S2). DAPC inferred the optimal number of genetic clusters as four, but each population contained components of each of these clusters (Fig. S3), and their relative frequencies in the populations did not show a geographical pattern.

#### Wintering populations

The proportion of genetic variation associated with the a priori defined wintering regions was not significantly different from zero for mtDNA or microsatellites (Table 2). There was also no evidence of significant substructure within wintering regions (both *P* > 0.15; Table 2). However, for mtDNA, we observed a global Φ_ST_ of 0.02 (*P* = 0.0156), and two of 66 pairwise comparisons were significantly different from zero (Table A4). If we excluded the Chinese and the Iranian population, the global Φ_ST_ value was reduced to 0.005, and no longer significantly different from zero (*P* = 0.15). For the microsatellite data, the overall population structure was very low but statistically significant (*F*_ST_ = 0.008; *P* = 0.0147), while none of the pairwise *F*_ST_ values were significant (Table A4). Exclusion of the Chinese and Iranian population resulted in an even lower *F*_ST_ of 0.004, which was not significantly different from zero (*P* = 0.17). No evidence of genetic substructure was detected by STRUCTURE (Fig. S2). DAPC indicated an optimal number of six clusters but, again, their relative frequencies within populations were not associated with geography (Fig. S3). The simulations performed in POWSIM showed that, with our specific microsatellite panel and sample sizes, we had high statistical power (>95%) to detect genetic substructure if the true *F*_ST_≥ 0.01 (Fig. S1).

## Discussion

### Genetic structure and lack of isolation by distance among breeding grounds

Our results provide no direct evidence of the four subpopulations of pochard resulting from migratory divides suggested by Blums and Baumanis (1990) based on analyses of ringing data. It may be not surprising that our phylogeographic and population genetics analyses did not provide support for these specific subdivisions given the difficulty of making quantitative inferences from the typically very limited amount of spatially heterogeneous ringing data (Hofer et al. 2006; Korner-Nievergelt et al. 2010). Nevertheless, our analyses show that breeding populations of pochard are not genetically homogenous across their very large distribution range. This is consistent with other species of migratory ducks where genetic structure was detected at different geographical scales and was sometimes suggested to be associated with recent colonization events (Gay et al. 2004; Tiedemann et al. 2004; Pearce et al. 2009). In pochard, genetic subdivision is likely caused by relatively recent processes given that clear phylogeographic patterns were absent and only allele or haplotype frequency based analyses detected relatively subtle signals of subdivision. This low level of structure among breeding populations is consistent with the high genetic diversity detected here, the large census size of approximately 1,500,000 pairs of the species (Scott and Rose 1996), its largely continuous breeding distribution, and high mobility.

It would be interesting in regard to the potential role of the species in the transmission of pathogens (see below) to investigate its movement patterns and the specific factors causing restricted gene flow between some of the breeding populations in more detail. Given that individuals are capable of traveling thousands of kilometers during annual migration, we would not expect physical restrictions to limit dispersal between breeding grounds. Indeed, the apparent absence of an isolation by distance (IBD) pattern between populations across Eurasia may suggest that dispersal occurs at least partially over very large distances as IBD can only build up if dispersal is spatially restricted (Slatkin 1993). Unfortunately, dispersal events between different breeding grounds are very rarely documented through ring recoveries, but anecdotal evidence suggests that long-distance dispersal occurs at least occasionally (Blums et al. 2002; our own unpub. data). Satellite tracking of individual ducks would provide very detailed information on dispersal rates and distances, but the associated costs typically limit such studies to a very small number of individuals (e.g., Gaidet et al. 2008).

It is worth noting that IBD in pochard may not have built up because populations are not at mutation–drift equilibrium, for example, because of a recent range expansion of the species. At a local scale, such an expansion has occurred. During the last centuries, changes in the availability of suitable water bodies and food sources have led to a westward extension of the species’ breeding range into Central and Western Europe (Cramp and Simmons 1977; Scott and Rose 1996) However, for the remaining distribution range, there are no historical records supporting the possibility of massive population increases and associated range expansions. This possibility requires dedicated further investigations and could have significant consequences for the transmission dynamics of associated pathogens (see e.g., Biek et al. 2007).

In this context, attention should be paid to potential sex differences in colonization and dispersal patterns of pochard given the higher level of genetic differentiation among breeding grounds in maternally inherited mtDNA than in biparentally inherited microsatellite markers (Table 2). Of course, this difference may be related to elevated rates of genetic drift in mtDNA as the effective population size of the mitochondrial genome is only one-fourth of the nuclear genome (Ballard and Whitlock 2004). Additionally, the homogenizing effect of male-biased dispersal would contribute to lower differentiation among populations in nuclear markers but not mtDNA (e.g., Petit et al. 2001; Ruedi et al. 2008). Male-biased dispersal and female philopatry in pochard, if indeed confirmed in future analyses, would be consistent with anecdotal evidence from ringing data that showed that, among the birds observed in two different breeding seasons, 78% of the females had returned to the same location (*N* = 18), compared to 33% of the males (*N* = 24; our own unpub. data). Along similar lines, Blums et al. (2002) estimated very high breeding-site fidelity rates of 0.88 for 1-year-old females and 1 for older females using long-term mark-recapture data. Similar sex-specific differences in the level of philopatry are reported from other migratory ducks (reviewed by Greenwood 1980; see also Robertson and Cooke 1999; Gay et al. 2004; Tiedemann et al. 2004; Hefti-Gautschi et al. 2009; Liu et al. unpubl. ms.). Breeding-site fidelity of females may be particularly important in species with uniparental offspring care such as pochard (Cramp and Simmons 1977; Scott and Rose 1996) because of the advantages of local experience in the choice of feeding and breeding sites (Greenwood 1980; Newton 2008).

### Population admixture at wintering grounds

Pochards sampled on the disjunct wintering grounds in Europe, the Caspian Sea, and eastern Asia show remarkably little genetic differentiation given the large geographical distances between them. Only population-based analyses including the wintering grounds in Asia revealed very weak signals of genetic subdivision. The latter result—taken alone—could be interpreted as reflecting a subdivision at least between the wintering grounds in Europe and East Asia at the western and eastern edges of the distribution range (Blums and Baumanis 1990). Indeed, this pattern is consistent with the general population structure detected among breeding grounds, where some significant pairwise *F*_ST_ values were observed between European and Asian populations (Table A4). More importantly, however, the much weaker genetic structure among wintering than among breeding grounds (Table 2) demonstrates extensive mixing of individuals from different origins during or after migration.

Given the very high levels of genetic diversity and relatively low genetic structure, it is not surprising that an aggregation of individuals from multiple breeding populations does not produce a classical population genetics signal for admixture among strongly subdivided populations such as elevated diversity or deviations from Hardy–Weinberg expectations (e.g., Wahlund effect; Wahlund 1928; reviewed by Manel et al. 2005). Large-scale admixture between wintering migratory ducks of the Northern Hemisphere is not unique to pochard as demonstrated by genetic analyses and satellite tracking for several species (Pearce et al. 2009; Liu et al. unpubl. ms.). Even ducks wintering several thousand kilometers apart, as for example in the case of northern pintail in Japan or North America, may be part of the same breeding population and even move between these wintering regions (Flint et al. 2009).

Extensive movements within and/or between winters are also the likely cause for the apparent genetic homogeneity among European wintering grounds of pochard. A subdivision was earlier proposed between a northwestern population wintering around the North Sea and a southeastern population wintering in central Europe and around the Mediterranean and Black Seas (Monval and Pirot 1989; Rose and Scott 1994). However, more recent ring recovery data indicate that individuals wintering in NW or SE Europe, respectively, may breed in the same areas extending into Western Siberia (Hofer et al. 2006), and that the two wintering populations overlap along the North Sea coast (Keller et al. 2009). Movements of pochard within a winter occur relatively frequently in response to environmental factors such as food availability and winter harshness (Keller et al. 2009), and may additionally promote contacts between individuals from different origins.

### Implications for AI transmission

The absence of a clear association between genetic variation and geography and, in particular, the apparent mixing of ducks on wintering grounds imposes strong limitations on further analyses of the transmission and spread of pathogens of pochard. H5N1-positive individuals from European wintering grounds shared mtDNA haplotypes with ducks from several continental regions, and genetic differentiation was insufficient to use individual-based assignment methods to trace the geographic origin of pochard with highly variable microsatellite genotypes. However, complete panmixia is apparently prevented by low levels of breeding philopatry, which indicates that even higher genetic resolution might allow the identification of the large-scale region of origin of individuals gathering on wintering grounds. Such an analysis would require hundreds or thousands of markers distributed across the entire genome, as recently exemplified in humans, which also show globally low genetic population structure (see Novembre et al. 2008). Additionally, the extensive collection of reference samples from the regions of interest would considerably improve the precision of such assignments (Manel et al. 2005).

It must be noted, however, that direct links between the movement of individuals or populations of pochard and the transmission and spread of HPAI H5N1 will be difficult to establish without considerably improved knowledge on the natural host spectrum, infection pathways, and the consequences of infection. H5N1-positive individuals were repeatedly found among pochard wintering in Europe but infections have also been detected in a number of other waterfowl species (Kim et al. 2009; Kou et al. 2009). Six published sequences of viral isolates from pochard are available from Switzerland and Germany, which were all assigned to the same viral subclade, 2.2.1 (Starick et al. 2007; Hofmann et al. 2008). However, this variant has also been detected in a range of other bird species, which limits the potential for strong associations between the virus and pochard in particular. Moreover, human activities (e.g., poultry trade) certainly contribute to the global spread of the virus without involvement of wild birds (e.g., Gilbert et al. 2006; Kilpatrick et al. 2006; Feare 2007; Gauthier-Clerc et al. 2007). A central question in the context of natural AI transmission is whether infected birds rapidly succumb to the disease or if they are still able to move while already excreting the virus. Infection experiments have demonstrated large differences between and within different species in this respect (reviewed in Gaidet et al. 2010). In pochard, four experimentally infected individuals showed mild to severe clinical symptoms while another four were asymptomatic but excreted the virus (Keawcharoen et al. 2008). While these results may not be directly transferable to wild birds, they still suggest that pochard cannot be ruled out as potential vectors of HPAI. Even if the course of the disease is severe, individual ducks may be able to travel long distances within the period of latency (Gaidet et al. 2008, 2010).

Continent-wide transport of HPAI by individual birds may be rather unlikely but transfer in a stepping-stone fashion through successively infected birds appears nevertheless possible (Reluga et al. 2007; Gaidet et al. 2010). In pochard, the observed very weak population subdivision even between East Asia and Europe and the large number of migrating individuals suggest that such processes could operate at an intercontinental scale. The likelihood for disease transmission and local spread may be particularly high on the wintering grounds, because of the increased stability of RNA viruses at low temperatures (Brown et al. 2009), the aggregation of birds from different geographic regions, and frequent movements within wintering areas.

## Data archiving

Data were deposited in the Dryad Repository: http://dx.doi.org/10.5061/dryad.vt642

## Appendix

**Table A1:** Sampling localities of the common pochard. Map ref. corresponds to numbers in Figure1. Coordinates, sample sizes for mtDNA (*N*_mt_) and nucDNA (*N*_nuc_) analyses, and migration status (breeding: June–August, wintering: November–February, and migrating: October or March) of each population are given

Map ref.	Location label	Location	Migration status	Country	Latitude	Longitude	*N*_mt_	*N*_nuc_
1	CZSb	Southern Bohemia	Breeding	Czech Rep.	49°08′30″N	14°43′38″E	24	29
2	CZDi	Divcice, Southern Bohemia	Breeding	Czech Rep.	49°06′33″N	14°18′32″E	9	20
3	SWSö	Södermanland	Breeding	Sweden	59°14′19″N	15°57′30″E	2	2
4	FISo	Southern Finland	Breeding	Finland	61°08′00″N	24°15′00″E	9	9
5	LAKa	Kanieris Lake	Breeding	Latvia	56°59′45″N	23°27′45″E	12	16
6	RUMo	Moscow region	Breeding	Russia	55°45′00″N	37°34′00″E	2	2
7	RUYe	Yekaterinburg region	Breeding	Russia	54°48′00″N	64°09′00″E	7	9
8	RUTo	Tomsk region	Breeding	Russia	56°27′00″N	84°57′00″E	1	1
9	RUTv	Tunka Valley	Breeding	Russia	51°50′00″N	102°22′00″E	6	6
10	RUBl	Selenga Delta, Baikal Lake	Breeding	Russia	52°22′05″N	106°32′45″E	21	21
11	CNQh	Qinghai Lake, Qinghai	Breeding	China	39°49′00″N	99°47′00″E	1	1
12	CNHa	Hasu Lake, Inner Mongolia	Breeding	China	40°36′00″N	110°57′00″E	4	1
13	CNQa	Qian'an, Jilin	Breeding	China	45°15′00″N	124°11′00″E	4	4
14	CNLf	Longfeng Lake, Heilongjiang	Breeding	China	46°30′19″N	125°10′43″E	16	12
15	CNXk	Xinkai Lake, Heilongjiang	Breeding	China	45°21′00″N	132°21′00″E	1	1
16	SPSe	Sevillia	Wintering	Spain	37°26′00″N	05°22′00″E	6	11
17	UKLe	Loch Leven	Wintering	United Kingdom	56°11′00″N	03°22′00″W	10	10
18	FRSa	Saint-Philbert-de-Grand-Lieu	Wintering	France	47°02′00″N	01°38′00″W	12	13
19	FRIn	Indre	Wintering	France	51°21′00″N	00°11′00″E	16	16
20	FRVa	Vanne	Wintering	France	47°37′00″N	05°49′00″E	1	1
21	FRCa	Camargue	Wintering	France	40°38′00″N	08°44′00″E	7	10
22	CHOb	Oberkirch	Wintering	Switzerland	47°09′00″N	08°06′00″E	24	67
23	CHNe	Neuhausen	Wintering	Switzerland	47°08′00″N	08°11′00″E	2	2
24	ITVa	Varese	Wintering	Italy	49°06′33″N	14°18′32″E	10	9
25	ITVe	Valle Zignago, Venezia	Wintering	Italy	45°40′00″N	12°53′00″E	13	12
26	DEBs	Lake Constance	Wintering	Germany	47°39′00″N	09°25′00″E	6	10
27	DEOs	Baltic Sea coast	Migrating	Germany	55°19′00″N	12°05′00″E	2	2
28	BEBr	Brussels region	Wintering	Belgium	50°43′00″N	04°29′00″E	10	10
29	SWSk	Skåne	Migrating	Sweden	55°59′59″N	15°11′40″E	1	1
30	BUBs	Black Sea coast	Wintering	Bulgaria	43°21′00″N	28°02′00″E	3	4
31	IRCs	Caspian Sea coast	Wintering	Iran	36°44′00″N	53°00′00″E	9	10
32	CNCh	Caihai, Guizhou	Wintering	China	26°51′22″N	104°13′09″E	21	21
33	CNQd	Qingdao, Shandong	Migrating	China	36°08′00″N	120°38′00″E	1	1
34	JPIz	Izunuma Lake	Wintering	Japan	38°42′00″N	141°08′00″E	1	1
Total							274	345

**Table A2:** Microsatellite loci and design of multiplex reactions: repeat motif, sequences of forward, F (fluorescently labeled) and reverse, R (pig-tailing: GTTTCTT added at 5′-end) primer, fluorescent dye, primer final concentration, and the original reference describing the primers are given for each locus

Mix	Locus	Repeat motif	Primer sequences (5'-3')	Dye	Concentration (μM)	Reference
	Caud13	(AC)n	F: ACAATAGATTCCAGATGCTGAA	PET	0.5	Huang et al. 2006
			R: ATGTCTGAGTCCTCGGAGC			
	MM07	(CA)n	F: GCAAAAGGGGCTATGAACAC	NED	1.25	Hefti-Gautschi & Koller 2005
			R: GGAGGCAGAGCTGGTTAGTG			
	Sfiμ3	(GA)nN_2_(GA)n	F: TATTCCAATATTCTGCAGGGAGG	6-FAM	1.25	Fields and Scribner 1997
			R: TCCAAGTTAATCAATTATCTGAT			
SET1	Smo11	(TG)nGA	F: AAATCAACCAAAGAGGCATAGCC	6-FAM	2.0	Paulus and Tiedemann 2003
			R: GCAGTTGTTTTGGAGGACAGACA			
	Sfiμ4	(GA)n	F: CTGAGGGGGAAGAGAATAAGAGA	PET	3.0	Fields and Scribner 1997
			R: CAGGGCAGTATTTTCAGGACATT			
	MM05	(AC)n	F: CCAAATCTGACCACCAGGAG	VIC	3.0	Hefti-Gautschi and Koller 2005
			R: GCCGTCAGGCAAATAGGAAC			
	CmAAT28	(AAT)n	F: TGAAAAGGGTCTTTACCTTATAT	6-FAM	2.0	Stai and Hughes 2003
			R: TTCCACATAAAAATTCATTCAGT			
	Apl12	(GA)n	F: AGTTGACCCTAATGTCAGCATC	6-FAM	1.25	Denk et al. 2004
			R: AAGAGACACTGAGAAGTGCTATTG			
	CmAAT38	(AAT)n	F: TCCCGAGCTACCACATTGAC	NED	3.0	Stai and Hughes 2003
			R: AGCAACTGGAAGGCATTTATCT			
	Smo4	(AG)nA	F: ACTTTCCACAGCCTCTTTCACAA	VIC	2.5	Paulus and Tiedemann 2003
			R: GACAGTGTTTGTCAATGGATTTT			
SET2	Aph13	(GA)n	F: CAACGAGTGACAATGATAAAA	6-FAM	1.25	Maak et al. 2003
			R: CAATGATCTCACTCCCAATAG			
	MM03	(CA)n	F: AAGTACATGTAAAAGCTGAAGTTGC	PET	3.0	Hefti-Gautschi and Koller 2005
			R: TTGCCTGATAAAAGGAATGC			
	Apl36	(CA)n	F: ATGCTTTGCTGTTGGAGAGC	NED	3.0	Denk et al. 2004
			R: TCCACTGGGTGCAAACAAG			
	Sfiμ2	(A)n(CA)n	F: CATAAACGGCTAATATGAAGTCT	6-FAM	2.0	Fields and Scribner 1997
			R: AGGCTAGATATTGCTCTTATCCT			

**Table A3:** Estimates of genetic variability at 12 polymorphic microsatellite loci in common pochard. For each population and each microsatellite locus, the sample size (*N*), the number of alleles (*N*_A_), the observed (*H*_O_) and the expected (*H*_E_) heterozygosity is indicated. The *H*_O_ values shown in bold indicate statistically significant deviations from Hardy–Weinberg equilibrium (HWE) based on 10,000 permutations

Pop	*N*	Parameter	Caud13	SMo11	Sfiμ04	MM05	CmAAT28	Apl12	CmAAT38	Smo4	Aph13	MM03	Apl36	Sfiμ02	Mean
CZSb	29	*N*_A_	7	4	2	12	10	7	7	3	8	4	2	3	5.750
		*H*_O_	**0.448**	0.517	0.138	0.862	1.000	0.655	0.552	0.069	0.724	0.276	0.345	0.138	0.477
		*H*_E_	0.682	0.516	0.128	0.868	0.846	0.715	0.700	0.067	0.707	0.303	0.328	0.131	0.508
CZDi	20	*N*_A_	5	4	3	10	9	6	5	1	8	4	4	1	5.000
		*H*_O_	**0.250**	0.600	0.200	0.900	0.800	0.350	0.350	0.000	0.550	0.333	0.600	0.000	0.493
		*H*_E_	0.539	0.536	0.261	0.831	0.845	0.684	0.411	0.000	0.555	0.444	0.446	0.000	0.469
FISo	9	*N*_A_	5	3	2	8	5	5	4	1	4	3	2	1	3.583
		*H*_O_	0.667	0.333	0.111	0.778	**0.111**	0.556	0.444	0.000	1.000	0.444	0.222	0.000	0.467
		*H*_E_	0.580	0.549	0.105	0.821	0.673	0.716	0.519	0.000	0.599	0.426	0.346	0.000	0.565
LAKa	16	*N*_A_	5	3	3	8	10	7	4	3	9	3	3	2	5.000
		*H*_O_	0.500	0.313	0.214	**0.813**	0.875	0.813	0.375	0.188	0.750	0.313	0.375	0.067	0.466
		*H*_E_	0.635	0.506	0.309	0.865	0.836	0.773	0.363	0.174	0.725	0.271	0.354	0.064	0.503
RUYe	9	*N*_A_	5	4	4	6	8	5	3	1	6	3	2	2	4.083
		*H*_O_	0.667	**0.444**	0.556	0.667	0.625	0.556	0.286	0.000	0.667	**0.167**	0.143	0.111	0.474
		*H*_E_	0.747	0.691	0.562	0.741	0.828	0.765	0.439	0.000	0.772	0.486	0.133	0.105	0.606
RUBl	21	*N*_A_	7	3	2	11	9	7	5	1	10	4	3	2	5.333
		*H*_O_	0.238	0.524	0.200	0.900	0.810	0.714	0.143	0.000	0.571	0.381	0.190	0.048	0.429
		*H*_E_	0.407	0.500	0.180	0.865	0.825	0.760	0.466	0.000	0.647	0.396	0.176	0.046	0.490
CNLf	12	*N*_A_	7	2	2	9	8	7	3	2	8	3	4	1	4.667
		*H*_O_	0.500	0.583	0.091	0.818	0.833	0.667	0.250	0.083	0.750	0.364	0.500	0.000	0.494
		*H*_E_	0.580	0.469	0.087	0.835	0.840	0.740	0.344	0.080	0.691	0.376	0.521	0.000	0.528
SPSe	11	*N*_A_	7	3	1	6	6	7	5	1	4	4	3	1	4.000
		*H*_O_	0.727	0.727	0.000	0.545	1.000	**0.545**	0.364	0.000	0.636	0.545	0.273	0.000	0.596
		*H*_E_	0.785	0.541	0.000	0.748	0.793	0.756	0.504	0.000	0.517	0.492	0.310	0.000	0.633
UKLe	10	*N*_A_	6	2	2	9	6	8	3	2	8	4	2	1	4.417
		*H*_O_	0.500	0.400	0.100	0.900	1.000	0.900	0.500	0.100	0.700	0.500	0.100	0.000	0.519
		*H*_E_	0.625	0.420	0.095	0.855	0.800	0.800	0.505	0.095	0.775	0.625	0.095	0.000	0.544
FRSa	13	*N*_A_	5	2	1	10	8	6	3	2	7	4	3	1	6.727
		*H*_O_	0.385	0.538	0.000	**0.769**	0.769	0.615	0.417	0.077	0.615	0.500	0.231	0.000	0.492
		*H*_E_	0.444	0.488	0.000	0.898	0.837	0.722	0.344	0.074	0.547	0.462	0.210	0.000	0.519
FRIn	16	*N*_A_	4	3	2	8	11	6	3	2	7	3	2	1	4.333
		*H*_O_	**0.250**	0.563	0.200	1.000	0.813	0.750	**0.188**	0.063	0.813	0.563	0.500	0.000	0.518
		*H*_E_	0.556	0.490	0.180	0.822	0.871	0.750	0.361	0.061	0.672	0.432	0.430	0.000	0.526
FRCa	10	*N*_A_	5	2	1	10	8	5	4	1	4	4	2	1	5.182
		*H*_O_	**0.200**	0.600	0.000	0.900	0.700	0.600	0.300	0.000	0.500	0.500	0.200	0.000	0.500
		*H*_E_	0.513	0.480	0.000	0.860	0.780	0.675	0.270	0.000	0.415	0.465	0.180	0.000	0.539
CHOb	67	*N*_A_	8	6	4	13	10	9	8	3	8	4	5	1	6.583
		*H*_O_	0.493	0.493	0.090	0.821	0.836	0.791	0.365	0.030	0.582	0.523	0.545	0.000	0.506
		*H*_E_	0.608	0.520	0.087	0.867	0.840	0.786	0.553	0.030	0.612	0.532	0.430	0.000	0.537
ITVa	9	*N*_A_	4	4	1	8	7	6	4	1	6	3	3	2	4.083
		*H*_O_	0.444	0.667	0.000	0.889	0.778	0.889	0.500	0.000	0.667	0.556	0.222	0.000	0.561
		*H*_E_	0.574	0.599	0.000	0.784	0.796	0.778	0.414	0.000	0.642	0.475	0.364	0.198	0.595
ITVe	12	*N*_A_	7	2	1	8	7	6	6	1	8	3	2	1	4.333
		*H*_O_	0.750	0.333	0.000	0.818	1.000	0.500	**0.455**	0.000	0.583	0.333	0.417	0.000	0.576
		*H*_E_	0.771	0.486	0.000	0.818	0.809	0.646	0.711	0.000	0.667	0.426	0.330	0.000	0.658
DEBs	10	*N*_A_	5	2	2	8	7	5	1	1	4	3	3	1	3.500
		*H*_O_	**0.500**	0.300	0.143	0.800	0.667	0.875	0.000	0.000	0.400	0.200	0.400	0.000	0.476
		*H*_E_	0.712	0.495	0.133	0.825	0.772	0.641	0.000	0.000	0.480	0.340	0.340	0.000	0.555
BEBr	10	*N*_A_	3	3	2	6	9	6	5	1	8	3	2	1	4.083
		*H*_O_	0.400	0.500	0.100	0.700	0.900	0.700	0.600	0.000	0.700	**0.200**	0.300	0.000	0.510
		*H*_E_	0.340	0.485	0.095	0.720	0.850	0.710	0.590	0.000	0.615	0.460	0.255	0.000	0.539
IRCS	10	*N*_A_	5	2	3	6	7	5	3	2	5	5	2	1	3.833
		*H*_O_	0.500	0.600	0.286	0.700	0.700	0.700	0.300	0.100	0.800	0.444	0.100	0.000	0.475
		*H*_E_	0.420	0.480	0.255	0.705	0.740	0.725	0.395	0.095	0.585	0.525	0.095	0.000	0.481
CNCh	21	*N*_A_	5	4	2	12	11	6	4	2	5	3	4	1	4.917
		*H*_O_	**0.381**	0.476	0.111	0.850	0.905	**0.619**	**0.474**	0.048	0.762	0.550	0.667	0.000	0.531
		*H*_E_	0.600	0.541	0.105	0.843	0.858	0.774	0.676	0.046	0.675	0.411	0.484	0.000	0.556

**Table A4:** Estimates of pairwise genetic differentiation among 19 populations of common pochard. Location abbreviations are given in Table A1, and are shown in bold for breeding populations. Above the diagonal are pairwise Φ_ST_ values for mtDNA and below the diagonal are pairwise *F*_ST_ values for microsatellites. Values shown in bold indicate significant genetic differentiation after Bonferroni correction

	**CZSb**	**CZDi**	**FISo**	**LAKa**	**RUYe**	**RUBl**	**CNLf**	SPSe	UKLe	FRSa	FRIn	FRCa	CHOb	ITVa	ITVe	DEBs	BEBr	IRCs	CNCh
CZSb	−	0.0697	**0.1328**	0.0773	0.0982	**0.0695**	**0.0743**	0.0349	0.0116	0.0503	0.0386	0.0502	**0.1281**	0.0466	0.0680	−0.0100	0.0684	0.0777	0.0202
CZDi	**0.0229**	−	0.1521	−0.0154	−0.0162	0.0196	0.0085	−0.0119	0.0144	0.0138	0.0389	0.0052	0.0262	0.0035	−0.0006	−0.0378	−0.0096	−0.0044	0.0306
FISo	0.0137	0.0239	−	0.1984	0.1942	**0.1718**	**0.1423**	0.1118	0.0726	0.1198	0.1437	0.1255	**0.2342**	**0.1728**	**0.1497**	0.0654	0.1638	0.1418	0.1259
LAKa	0.0082	0.0183	0.0088	−	−0.0076	0.0242	−0.0011	0.0417	0.0053	0.0356	−0.0050	0.0654	0.0396	0.0131	−0.0189	−0.0304	−0.0338	−0.0152	0.0257
RUYe	**0.0363**	**0.0357**	0.0299	0.0194	−	0.0604	0.0263	0.0256	0.0151	0.0578	0.0072	0.0605	0.0365	0.0026	0.0268	−0.0044	0.0196	––0.0013	0.0426
RUBl	**0.0205**	0.0148	0.0167	0.0219	**0.0542**	−	−0.0016	0.0167	0.0214	0.0308	0.0457	0.0322	**0.0455**	**0.0787**	−0.0219	−0.0507	−0.0144	0.0155	0.0342
CNLf	0.0036	−0.0038	0.0097	0.0047	0.0301	0.0089	−	−0.0219	0.0068	0.0113	0.0206	0.0288	0.0133	0.0309	−0.0176	−0.0571	−0.0214	−0.0270	0.0376
SPSe	0.0182	0.0142	0.0080	0.0112	0.0184	0.0323	0.0148	−	0.0137	0.0455	0.0126	0.0387	0.0494	0.0151	0.0240	−0.0574	−0.0028	−0.0146	0.0478
UKLe	0.0006	0.0200	0.0104	0.0051	0.0249	0.0005	−0.0082	0.0098	−	−0.0233	−0.0258	−0.0347	0.0537	−0.0140	−0.0005	−0.0700	0.0105	−0.0070	−0.0085
FRSa	0.0144	−0.0048	0.0190	0.0152	0.0536	−0.0044	−0.0078	0.0088	−0.0139	−	0.0220	−0.0312	0.0380	0.0199	0.0037	−0.0471	0.0441	0.0075	0.0273
FRIn	**0.0270**	0.0020	0.0085	0.0144	0.0211	0.0231	0.0018	0.0133	0.0229	0.0103	−	0.0262	**0.0767**	0.0141	0.0075	−0.0533	0.0052	0.0101	−0.0020
FRCa	**0.0305**	0.0078	0.0514	0.0344	**0.0710**	0.0103	−0.0020	0.0365	0.0099	−0.0213	0.0325	−	0.0319	0.0284	0.0176	−0.0350	0.0425	0.0164	0.0460
CHOb	0.0066	0.0072	0.0117	**0.0150**	**0.0366**	0.0151	−0.0065	0.0081	−0.0036	−0.0011	0.0129	0.0143	−	0.0092	0.0257	0.0160	0.0371	0.0032	0.1067
ITVa	−0.0020	−0.0024	−0.0009	−0.0139	0.0113	0.0116	−0.0129	−0.0134	−0.0116	−0.0102	−0.0104	0.0048	−0.0116	−	0.0412	−0.0121	0.0515	−0.0011	0.0496
ITVe	−0.0045	0.0131	0.0309	0.0233	0.0311	0.0293	0.0086	0.0059	0.0012	0.0164	0.0263	0.0176	−0.0001	−0.0068	−	−0.0628	−0.0410	−0.0063	0.0215
DEBs	0.0241	0.0090	0.0375	0.0071	0.0453	0.0086	−0.0044	0.0080	0.0169	0.0087	0.0148	0.0050	0.0169	0.0001	0.0302	−	−0.0526	−0.0422	−0.0372
BEBr	−0.0030	0.0005	0.0177	0.0186	0.0426	0.0010	−0.0122	0.0231	−0.0061	−0.0142	0.0166	−0.0027	−0.0079	−0.0145	−0.0027	0.0213	−	−0.0115	0.0232
IRCs	**0.0317**	0.0080	0.0132	0.0153	0.0117	0.0103	0.0202	0.0198	0.0124	0.0036	0.0065	0.0309	0.0215	−0.0057	0.0519	0.0404	0.0046	−	0.0411
CNCh	0.0041	0.0050	0.0169	**0.0223**	0.0174	**0.0247**	−0.0043	0.0268	0.0120	0.0172	0.0211	0.0227	0.0049	0.0010	−0.0017	0.0400	−0.0008	0.0250	−

## Supporting information

**Figure S1.** Analysis of statistical power to detect significant differentiation between pochard populations based on the 12 microsatellite markers used in this study.

**Figure S2.** Analyses with the program STRUCTURE v.2.3.1 provide no evidence of genetic substructure within (A) breeding or (B) wintering pochard.

**Figure S3.** Inference of genetic clusters using discriminant analysis of principal components (DAPC) for breeding (A) and wintering (B) populations of common pochard, respectively.
