# Supplementary material for: Range-wide genetic population structure of common pochard (Aythya ferina): a potentially important vector of highly pathogenic avian influenza viruses
Source: Ecol Evol. 2011 Dec;1(4):529–45. doi: 10.1002/ece3.46 (PMC3287338; doi:10.1002/ece3.46)

Figure A1 Analysis of statistical power to detect significant differentiation between pochard populations based on the 12 microsatellite markers used in this study. Datasets with six pre-defined levels of population differentiation (F_{ST} values of 0.001, 0.0025, 0.005, 0.01 and 0.02) were generated using the program POWSIM. Statistical power was defined as the proportion of times the null hypothesis of equal allele frequencies across populations was rejected using a chi-square test or a Fisher's exact test. (Br = breeding populations; Win = wintering populations).

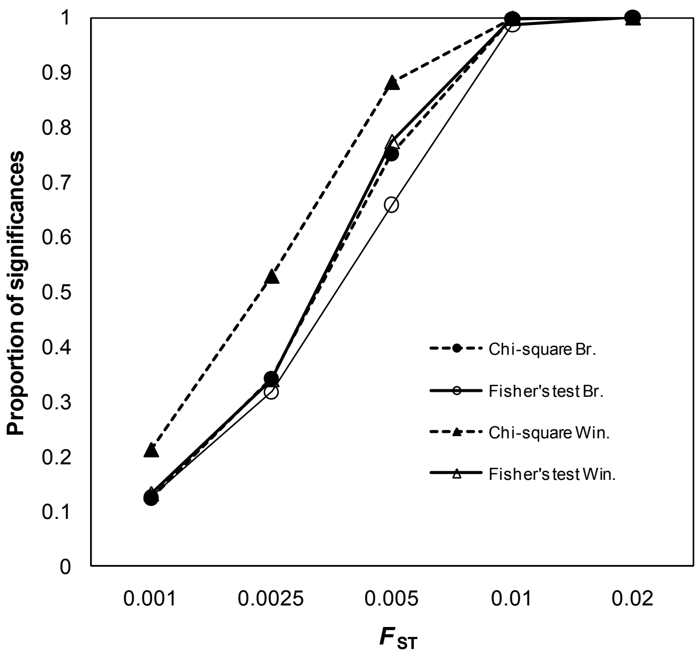

Supplement: Supplementary file 1 [file ece30001-0529-SD1.pdf]
