# Supplementary material for: Range-wide genetic population structure of common pochard (Aythya ferina): a potentially important vector of highly pathogenic avian influenza viruses
Source: Ecol Evol. 2011 Dec;1(4):529–45. doi: 10.1002/ece3.46 (PMC3287338; doi:10.1002/ece3.46)

**Figure A2** Analyses with the program STRUCTURE v.2.3.1 provide no evidence of genetic substructure within (a) breeding or (b) wintering pochard. Shown are the means of the posterior probability Ln P (D) (± *SD*) for different numbers of genetic groups (*K*) and averaged over 10 runs per K.

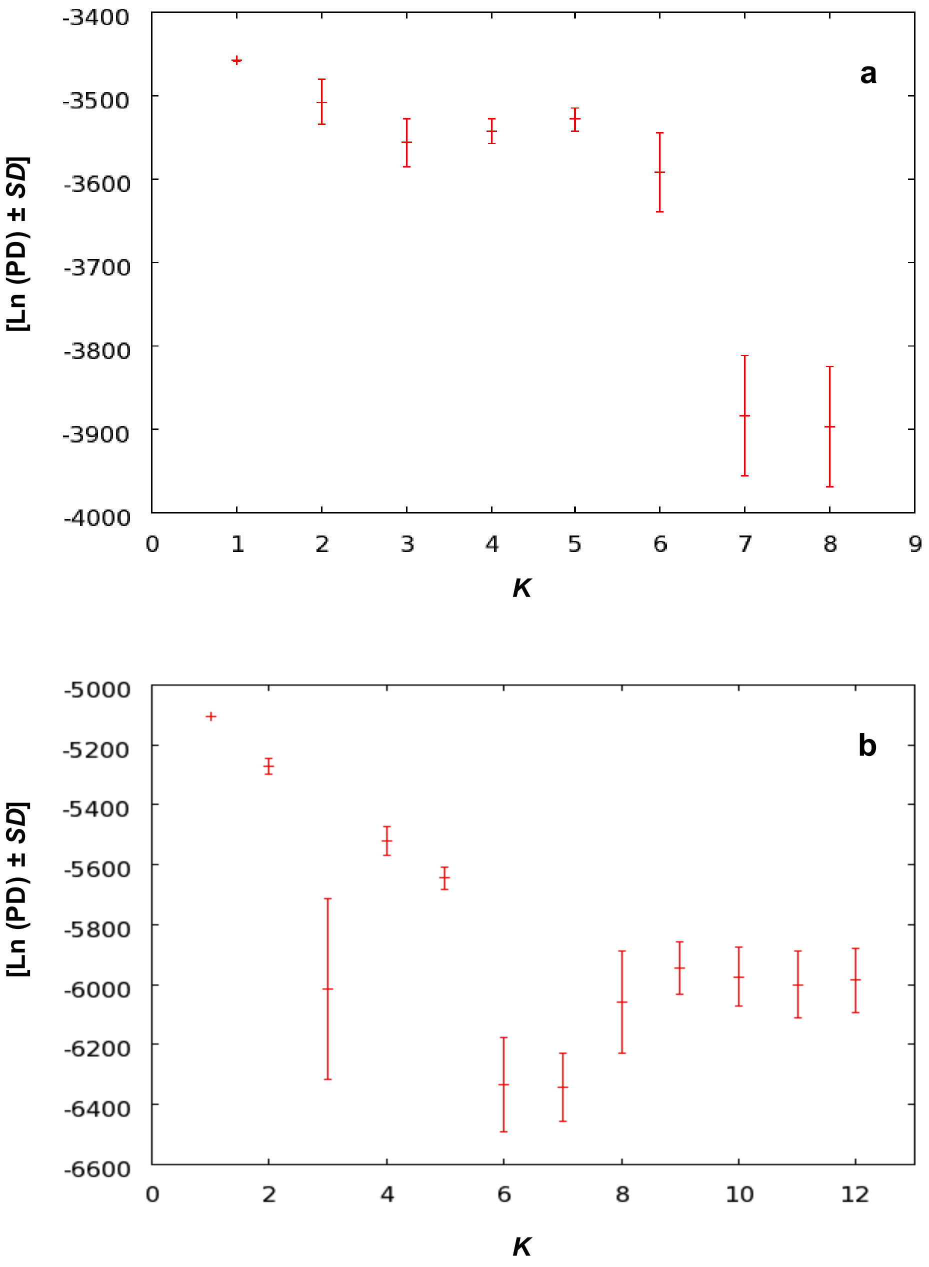

Supplement: Supplementary file 2 [file ece30001-0529-SD2.pdf]
