# Supplementary material for: Range-wide genetic population structure of common pochard (Aythya ferina): a potentially important vector of highly pathogenic avian influenza viruses
Source: Ecol Evol. 2011 Dec;1(4):529–45. doi: 10.1002/ece3.46 (PMC3287338; doi:10.1002/ece3.46)

**Figure A3** Inference of genetic clusters using discriminant analysis of principal components (DAPC) for breeding (a) and wintering (b) populations of common pochard, respectively. In all analyses of DAPC, 33 principal components (PCs) were retained in the data transformation step which accounted for more than 90% of the total genetic variability. In each representation, the bottom left plot shows the Bayesian information criterion (BIC) for different numbers of clusters (*K*), with the optimal *K* indicated by a red circle. The scatter plots demonstrate the first two principal components of the DAPC for the optimal *K*. The inferred genetic clusters are displayed by differently coloured inertia ellipses with dots representing individuals. The barplots illustrate the proportion of individuals from each population assigned to each of these genetic clusters.

(a)

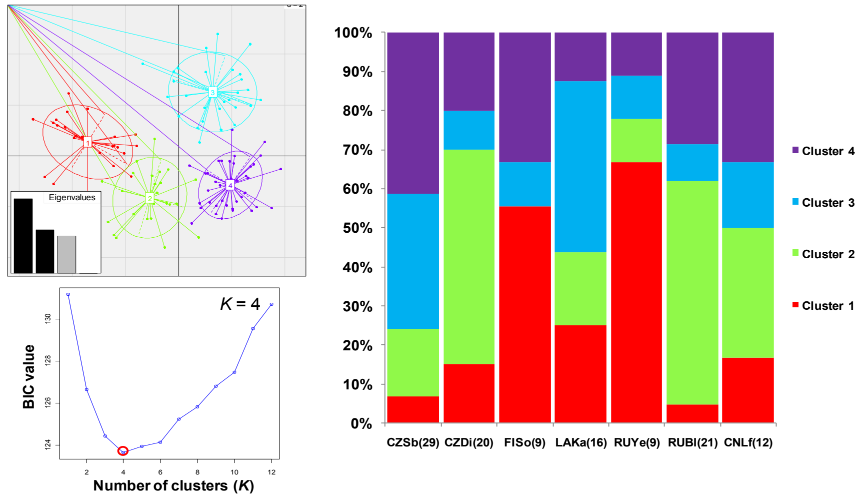


(b)

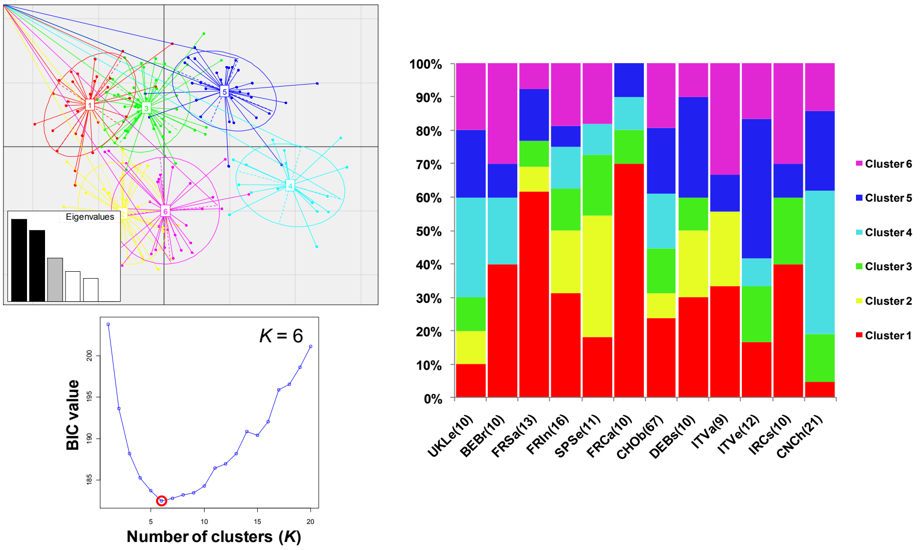

Supplement: Supplementary file 3 [file ece30001-0529-SD3.pdf]
